# Intra-Cochlear Current Spread Correlates with Speech Perception in Experienced Adult Cochlear Implant Users

**DOI:** 10.3390/jcm10245819

**Published:** 2021-12-13

**Authors:** Charles-Alexandre Joly, Pierre Reynard, Ruben Hermann, Fabien Seldran, Stéphane Gallego, Samar Idriss, Hung Thai-Van

**Affiliations:** 1 Institut de l’Audition, Institut Pasteur, Université de Paris, INSERM, 75012 Paris, France; charles-alexandre.joly@chu-lyon.fr (C.-A.J.); pierre.reynard@chu-lyon.fr (P.R.); 2 Université Claude Bernard Lyon 1, 69100 Villeurbanne, France; ruben.hermann@chu-lyon.fr (R.H.); sgallego@hotmail.fr (S.G.); 3 Service d’Audiologie et d’Explorations Otoneurologiques, Hôpital Edouard Herriot, Hospices Civils de Lyon, 69003 Lyon, France; samar.idriss@chu-lyon.fr; 4 Integrative, Multisensory, Perception, Action and Cognition Team (IMPACT), Inserm U1028, CNRS UMR5292, Lyon Neuroscience Research Center, 69675 Bron, France; 5 Service d’ORL, Chirurgie Cervico-Faciale et d’Audiophonologie, Hospices Civils de Lyon, Hôpital Edouard Herriot, 69003 Lyon, France; 6 MED-EL GmbH, CS 70062, 06902 Sophia Antipolis, France; fabien.Seldran@medel.com; 7 Neuronal Dynamics and Audition Team (DNA), Laboratory of Cognitive Neuroscience, CNRS UMR7291, Aix-Marseille University, CEDEX 3, 13331 Marseille, France

**Keywords:** cochlear implant, intra cochlear current spread, speech intelligibility, channel interaction

## Abstract

Broader intra-cochlear current spread (ICCS) implies higher cochlear implant (CI) channel interactions. This study aimed to investigate the relationship between ICCS and speech intelligibility in experienced CI users. Using voltage matrices collected for impedance measurements, an individual exponential spread coefficient (ESC) was computed. Speech audiometry was performed to determine the intelligibility at 40 dB Sound Pressure Level (SPL) and the 50% speech reception threshold: I40 and SRT50 respectively. Correlations between ESC and either I40 or SRT50 were assessed. A total of 36 adults (mean age: 50 years) with more than 11 months (mean: 34 months) of CI experience were included. In the 21 subjects for whom all electrodes were active, ESC was moderately correlated with both I40 (r = −0.557, *p* = 0.009) and SRT50 (r = 0.569, *p* = 0.007). The results indicate that speech perception performance is negatively affected by the ICCS. Estimates of current spread at the closest vicinity of CI electrodes and prior to any activation of auditory neurons are indispensable to better characterize the relationship between CI stimulation and auditory perception in cochlear implantees.

## 1. Introduction

Cochlear implants (CI) are implantable biomedical devices used for auditory rehabilitation among patients with severe to profound sensorineural deafness.

The CI audio processor performs a spectral decomposition of the signal into different frequency bands or channels, the number of which varies from 12 to 24 depending on the CI system. The intensity modulation of each acoustic band is converted into electrical amplitude modulation and then transmitted to an electrode array which is equipped with as many electrodes as channels generated by the processor. With respect to cochlear tonotopy, the electrical stimulation of each channel is distributed to a unique electrode according to its insertion depth in the cochlea. Each electrode is assumed to stimulate its own distinctive population of auditory nerve fibres. Hence, tonotopy is partially restored supplying sufficient auditory information and allowing implanted patients to perceive and understand speech from one interlocutor in a quiet environment.

**Figure 1:**
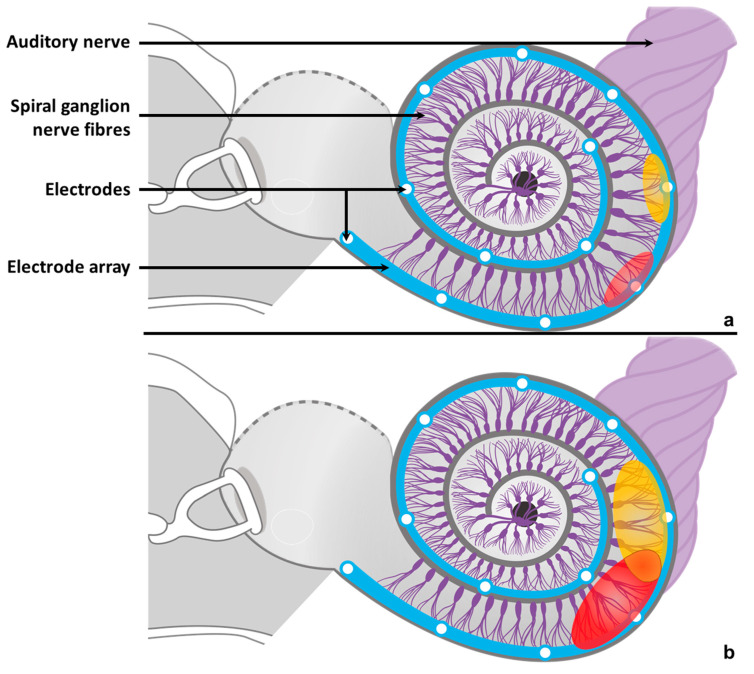
Examples of interactions induced by the intracochlear spread of current from the cochlear implant. Whenever the cochlear implant’s electrode array delivers electrical stimulations (red and yellow areas), the current spreads from each activated electrode to the surrounding neural tissue. If the spread is limited (**a**), each electrode will stimulate a distinct part of the neural tissue and thus evoke its own auditory sensation independent of the others. For a larger current spread (**b**), a shared part of neural tissue is stimulated by adjacent electrodes at the same time with a higher risk of channel interaction (orange area), and distinguishing sounds evoked by the interacting electrodes may become more difficult.

However, wide and unpredictable disparities in hearing capacities persist across CI users, particularly for competitive listening situations, e.g., when speech perception is challenged by the presence of noise. Two principal factors are thought to explain such variations. On the one hand, the status of pre-synaptic, synaptic, and post-synaptic functions at the level of the auditory end-organ has been incriminated [1,2]. This factor relates to deafness aetiology as well as demographic data including deafness onset or duration, and age at implant [3,4]. However, such demographics can only explain 20% of variance in auditory performance across subjects [5,6]. On the other hand, the transmission quality of the electrical stimulation is likely to be involved [7,8,9,10]. Its efficiency depends on various parameters: stimulation mode, spacing between the implant’s electrodes, distance between electrodes and cochlear nerve fibres, and electrodes’ surface and impedance. The latter reflects the capacity of CI electrodes to correctly deliver the electrical signals encoded by the CI processor. To obtain accurate and efficient stimulation of the auditory pathway, it is preferable to have a low and homogenous distribution of impedances along the electrode array. The neural stimulation provided by a CI cannot therefore be as focal as that induced by acoustical signals in normal hearing subjects. In the absence of direct contact between the electrodes and the cochlear spiral ganglion nerve fibres, the current will spread from each activated electrode through the surrounding tissue, and part of that current will then stimulate the nerve fibres (Figure 1). Although the intracochlear current spread (ICCS) may vary across subjects, it leads to interaction phenomena between adjacent electrodes, i.e., adjacent electrodes are likely to stimulate common parts of the neural tissue and thus produce either a facilitation [11] or a limitation effect due to the neural refractory period. Channel interactions result from the combination of both phenomena [12] and lead to a number of independent channels available for the cochlear implantee lower than the actual number of activated electrodes [13], resulting in difficulties in processing multiple frequencies simultaneously. Accordingly, such interactions may affect auditory perception and explain why increasing the number of stimulating electrodes never allows performance improvement [14,15,16].

In recent years, there has been growing interest in assessing interactions between CI electrodes using approaches based on the neural tissue’s own electrophysiological response [17,18,19,20] and/or psycho-acoustical testing [21,22,23,24,25]. Earlier research mainly focused on the spread of excitation (SOE) of the electrically evoked compound action potential (eCAP). Specifically, the eCAP SOE approach used the intracochlear electrode array for measuring the amplitude of the endo-cochlear neural response as a function of distance from the stimulating electrode. However, while both electrophysiological and psycho-acoustical measures of channels’ interaction require effective neural activation, data related to ICCS from each activated CI electrode upstream of the targeted neural tissue remain scarce, despite the growing interest in it. Up to now, most of the studies about ICCS used computational models [26,27] or a physical model [28] to characterize the ICCS according to electrode array placement or the stimulation strategies. Only a few studies evaluated the relationships between ICCS and speech perception [27] or ICCS and eCAP SOE [29]. To further explain heterogeneity in auditory performance across experienced CI users, it may also be useful to assess ICCS at the level of the stimulating electrode, i.e., even before the response of the surrounding neural tissue is triggered.

**Figure 2:**
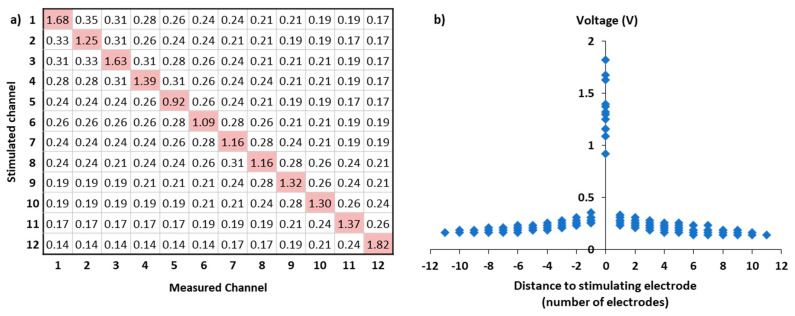
Example of the voltage matrix, in V (**a**) of one subject (S35) collected during impedance field telemetry measurement with the MAESTRO software (MED-EL GmbH, Innsbruck, Austria). Measurement of electrodes’ impedance consists in activating one electrode at a time and then measuring the voltage detected by every electrode of the cochlear implant array. The resulting voltages are plotted as a function of the distance between the stimulating and the recording electrode expressed in number of electrodes (**b**).

Along the intracochlear electrode array, electrode impedance can be determined based on voltage electrodes: after the emission of a calibrated stimulation by the channel of interest, voltage is indeed measured by all electrodes. These measures can be plotted as a function of the distance between stimulating and recording electrodes and expressed in a voltage matrix (Figure 2). Since, for a given CI system, the characteristics of electrical stimulation are constant across electrodes, any changes in voltage electrodes also reflect variations in ICCS [30,31,32]. Voltage measurements can thus be used to estimate ICCS in every cochlear implantee.

The main purpose of this study was to investigate to what extent ICCS can cause inter-channel interaction and then affect speech perception. Specifically, in a population of experienced adult CI users, the study aimed to (i) use the voltages collected for impedance computation to carry out ICCS measurements (ii) investigate the correlation between ICCS and speech perception scores.

## 2. Materials and Methods

### 2.1. Subjects

Only adults (18+) with active CI for at least 11 months were recruited at the Edouard Herriot university hospital in Lyon, France. Every participant was implanted with a MED-EL (Innsbruck, Austria) device.

The study was approved by the regional ethics committee (CPP 15/069). Written consents were obtained for all participants.

### 2.2. Procedure

For each subject, data were collected during one of the visits scheduled annually to assess CI function and, if necessary, to adjust the fitting parameters.

#### 2.2.1. Speech Recognition Tests

Speech recognition was evaluated by experienced audiologists specialized in CI using disyllabic words [33]. Each test list contains 10 disyllabic words pronounced by a masculine voice with constant interval. Subjects were asked to repeat the words and responses were counted in a binary way: they were considered false as soon as a part of the word was wrongly repeated.

Six test lists were delivered in free field in silence in a soundproof booth. Words were administered through a speaker situated on meter in front of the subject and controlled by an audiometer (Madsen Orbiter 922, GN Otometrics, Taastrup, Denmark). Each list was played at a constant presentation level. The presentation intensity varied between lists from 30 to 80 dB Hearing Loss (HL) with a 10 dB HL increment.

The scores obtained at each presentation level were used to compute the 50% speech reception threshold (SRT50) which is the stimulation level required to reach 50% of correct repetitions. For each subject, the SRT50 and the intelligibility measured at 40 dB HL (I40) were collected for analysis. The latter variable was selected because it had a broader variance compared to the score obtained with other presentation levels, indicating that I40 had the highest discriminatory power.

#### 2.2.2. Impedance and Field Telemetry (IFT) Recording and Exponential Spread Coefficient (ESC) Computation

**Figure 3:**
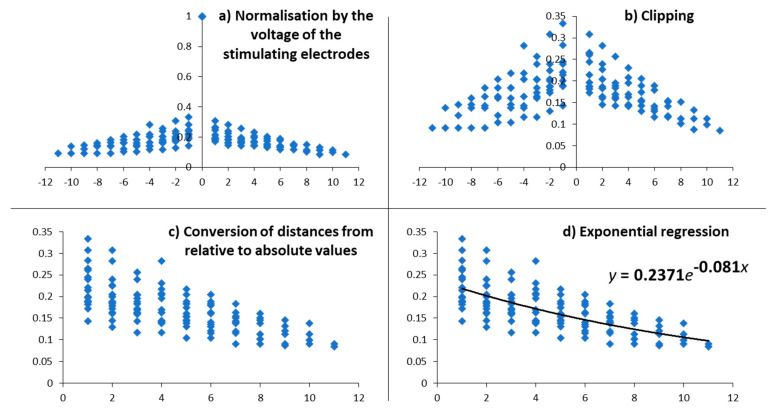
Example of the data processing performed for the voltages measured in the subject S35. The normalised voltages are expressed in arbitrary unit, the distance between the stimulating and the recording electrode is expressed in number of electrodes. (**a**) For each stimulating electrode, the voltages of all active electrodes are normalised according to the voltage measured at the level of the stimulating one. (**b**) The normalised voltages of the stimulating electrodes (all equal to 1) are removed from the data. (**c**) As we consider here the current spread to be symmetrical, the relative distances are converted in absolute values. (**d**) An exponential regression was computed to determine an exponential spread coefficient (ESC, in exponent) reflecting the decrease in current as the distance from the stimulation increases. We use this coefficient as a measure of the current spread with the assumption that a broader spread will result in a lower voltage decrease with distance and thus a higher coefficient (close to 0), and conversely, a narrower spread will result in a lower coefficient.

Impedances were recorded for each electrode using the clinical software (Maestro) and interface (MAX) provided by the CI manufacturer (MED-EL, Innsbruck, Austria). As some electrodes were inactivated, corresponding data were removed from the dataset, only significant values remained. For each stimulating electrode, the voltages of all active electrodes (including the stimulating one) were normalised by the voltage measured at the level of the stimulating electrode (Figure 3a). The normalised voltages of the stimulating electrodes were removed (Figure 3b). We converted relative distances between electrodes (in number of electrodes) to absolute values (Figure 3c). Then, an exponential regression was computed to fit the normalised voltages as a function of the absolute distance to the stimulating electrodes (Figure 3d).

The equation of the regression was:

V(*x*) = V_max_ × e*^αx^*

where *x* is the absolute distance between the stimulating electrode and the recording ones, in number of electrodes; V(*x*) is the mean voltage measured *x* electrodes further away from the stimulating one, in Volts; V_max_ is a constant and corresponds to the theoretical maximal voltage associated to the stimulating electrodes, in Volts; and *α* is the ESC which transcribes the voltage attenuation as a function of absolute distance to the stimulating electrode and so is expected to be negative. The lower the current spread, the higher the slope, the more negative the ESC becomes.

### 2.3. Statistical Analyses

Statistical analyses were carried out in subjects for whom ESC, SRT50, and I40 could be obtained. Statistical analyses were performed using IBM SPSS (IBM Corp. Released 2015. IBM SPSS Statistics for Windows, Version 23.0. Armonk, NY, USA: IBM Corp.) and the significance threshold was set at 0.05. After assessing the normality of the data using Shapiro-Wilk tests, the Bravais-Pearson correlation test was used to evaluate the correlations between ESC and SRT50, as well as between ESC and I40. The strength of the correlations was categorised according to Chan’s classification [34].

## 3. Results

### 3.1. Feasibility

Thirty-six adults (22 females, 14 males) were recruited. The mean age at implantation was 46.8 (±16.8) years. At the time of testing, participants (mean age 49.8 ± 17.5 years) had between 11 months and 10 years (mean: 34.4 ± 29.2 months) of CI experience. Demographic data for each subject are presented in Table 1.

All channels (12/12) could be activated in 24 out of 36 subjects. In the remaining 12 subjects, between 8 and 11 electrodes were activated. A total of 408 channel electrodes (94.4%) were activated. Inactivated electrodes were excluded from analyses prior to ESC computation. The number and reasons for CI electrode inactivation are indicated in Table 1.

Voltage matrices were successfully obtained using IFT, allowing ESC to be calculated for every participant. SRT50 was not computable in 3/36 subjects. Two of them never reached 50% of speech recognition (S-06, S-29). The third never scored less than 50% even for the minimal testing level (S-32). Hence, these three subjects were not considered for further analysis.

### 3.2. Correlations between ESC and Speech Recognition

Statistical analyses were conducted in two groups. The first group included all participants (*n* = 33). In subjects having part of the electrode array inactivated, the number of available channels is diminished resulting in an increase in the stimulate rate for the remaining electrodes. Since speech perception abilities may vary both with the number of active channels and the stimulation rate, a second analysis was conducted among participants with a fully activated electrode array (*n* = 21).

#### 3.2.1. Among All Subjects with Measurable SRT50

Among the 33 subjects with computable SRT50, the mean SRT50 was 41.4 dB (±7.3) and mean I40 was 47% (±25).

**Figure 4:**
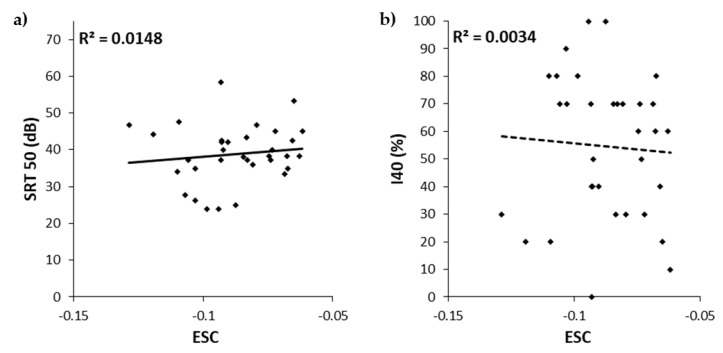
Scatter plots of the 50% speech reception threshold (SRT50, (**a**)) and the intelligibility at 40 dB (I40, (**b**)) as a function of the exponential spread coefficient (ESC) for all 33 participants. Bravais-Pearson determination coefficients are indicated in the upper left corner.

No correlation was found neither between ESC and SRT50 (r = 0.122, *p* = 0.5), nor between ESC and I40 (r = −0.059, *p* = 0.745; Figure 4).

#### 3.2.2. Among Subjects with Measurable SRT50 and All Electrodes Activated

Among the 21 subjects with 12/12 activated electrodes, the mean SRT50 was 37.3 dB (±7.6) and mean I40 was 59% (±25).

**Figure 5:**
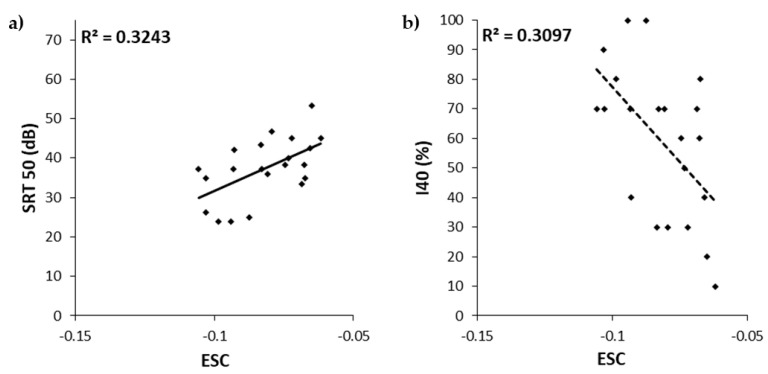
Scatter plots of the 50% speech reception threshold (SRT50, (**a**)) and the intelligibility at 40 dB (I40, (**b**)) as a function of the exponential spread coefficient (ESC) in the 21 subjects with all electrodes activated. Bravais-Pearson determination coefficients are indicated in the upper left corner.

A moderate significant correlation was observed between both ESC and SRT50 (r = 0.569, *p* = 0.007), and between ESC and I40 (r = −0.557, *p* = 0.009; Figure 5). These results show that as the ESC decreased, SRT50 decreased and I40 increased. The ESC variability was found to explain 32% and 31% of the SRT50 and I40 variability, respectively.

## 4. Discussion

The present study assessed the relationships between speech perception abilities and ICCS in experienced adult CI users. Here, ICCS was estimated using an intra-cochlear measure computed from the voltage matrices collected for impedance recording. The resulting index, ESC, was found to be strongly correlated with speech perception measurements in subjects who were benefiting from an activation of all the channels. For them, ESC was positively correlated with SRT50 and negatively correlated with I40. These results indicate that, with the same number of channels, broader ICCS is associated with weaker speech perception abilities.

Since the development of multi-channel CI in the late 1990s, researchers have investigated the potential impact of channel interaction on speech perception abilities in cochlear implanted subjects. Pioneer studies used psycho-acoustical tests to evaluate such interactions, including electrode discrimination and forward-masking, but led to divergent results. For instance, Zwolan et al. [25] did not find any correlation between electrode discrimination and speech perception but observed that inactivation of non-discriminable electrodes led to better speech perception. While Hughes and Abbas [23] did not find any significant correlation either, two studies showed that better electrode discrimination was associated with better speech perception, in children [21] and adults [22]. In CI subjects, forward masking evaluates the ability of an electrical stimulation to mask the perception of a second stimulus presented shortly after. Throckmorton and Collins [24] found that forward-masking, electrode discrimination, and speech perception are correlated with each other. These authors demonstrated that the ability to discriminate between adjacent electrodes is a predictor of speech perception.

In the early 2000s, implementation of reverse telemetry allowed systematic impedance recording and eCAP measurements. Easy and fast recording of eCAPs, compared to other electrophysiological tests, permitted intensive investigations of its clinical usefulness. Many efforts were made to predict CI fitting parameters and auditory performance of CI users from eCAP measures [35,36,37,38,39]. In this context, one eCAP acquisition method measuring the spread of neural excitation, the so-called SOE, was thought to be of potential interest for objective evaluation of channel interactions.

The eCAP SOE referred to two different techniques. According to the first technique, the amplitude of auditory nerve fibres’ responses is measured as a function of the distance between stimulating and recording electrodes. With the second technique, two different electrodes act as marker and probe so that an electrophysiological forward-masking paradigm can take advantage of the nerve refractory period [40]. Doing so, eCAP amplitude is expected to decrease as the distance between the two electrodes used as masker and probe increases. The advantage of SOE forward masking paradigm over psycho-acoustical testing is the removal of any interference due to attention related-factors. Strong correlations were found between electrophysiological and psycho-acoustical forward masking tests [20]. However, using the two SOE recording techniques in six subjects, Cohen et al. reported that neither psycho-acoustical forward-masking nor SOE were related to speech performance [17]. Operating with a modified SOE algorithm which gave narrower spread function, the same authors observed that the spread still remains broader with eCAP SOE—compared to psycho-acoustical testing-based measurement [18]. They suggested that eCAP SOE merely reflects neuronal current diffusion through the cochlear tissues and fluids separating the spiral ganglion neurons from the electrode array, rather than the actual number of excited neurons. While some authors did not find any correlation between SOE and speech perception [41,42], others reported a significant relationship with vowel identification provided SOE was evaluated globally for the entire electrode array rather than at individual electrode level [43]. Scheperle and Abbas [44] compared SOE and auditory cortical responses for different levels of channel interaction obtained by varying CI fitting parameters. They observed that peripheral (SOE) and central responses were not correlated with each other, which may explain why single electrode-SOE is so poorly correlated with speech perception performances.

Using electrically evoked auditory brainstem response (eABR), Guevara et al. [19] proposed more recently a new technique for estimating channel interaction. This technique consisted of comparing eABR amplitude resulting from the simultaneous stimulation of four electrodes, to the sum of eABR amplitude calculated for each individual electrode. The ratio of the two resulting measures was found to be correlated with phonemic discrimination in CI users.

All the studies above assessed the relationship between speech perception abilities and either a psycho-acoustical or electrophysiological measure which requires effective activation of the afferent auditory pathway. However, before stimulating the spiral ganglion neurons, the CI electrical current must flow through cochlear fluids and tissues.

So far, only very few studies have estimated ICCS at the closest vicinity of the CI electrodes’ surface or investigated its influence on speech perception performance. Using clinical observations from 14 CI adult subjects, Jürgens et al. [27] proposed a predictive model of speech-in-noise based on ICCS and “internal noise”—representing both language skills and anamnesis—as regressors. In their study, a text reception threshold was used to determine language skills, while the patient anamnesis was used to compute an auditory performance score based on deafness duration and aetiology, use of hearing aid prior to implantation, and age at implant. Only internal noise, determined as such, was a significant predictor of speech-in-noise in individual subjects. However, speech perception abilities were found to diminish when ICCS increased at the group level. Accordingly, the present study also found an association between ICCS and speech perception in CI users.

The role of patient anamnesis in post-implant auditory outcomes has been extensively investigated in the literature [5,6,45,46]. Using multifactorial predictive models, previous works were able to explain up to 25% of the variability in hearing outcomes [6]. Interestingly, the ICCS alone explained herein around 30% of speech intelligibility variability in a group of adult CI users, when the number of activated channel electrodes was maximal. Yet, this was not the case when the subjects with one or more inactive channels were also considered. Although statistical analysis was prevented by the low number of patients in each group, the results herein show that SRT50 tended to be higher and I40 lower in the group including subjects with a partly activated electrode array. It is likely that channel inactivation also impacted speech perception abilities in the latter group, making the role of ICCS per se more difficult to pinpoint.

The ICCS is the most peripheral electrical phenomenon that could affect both integrative psycho-acoustical and electrophysiological measures. This may explain why previous attempts to predict hearing perception from electrophysiological measures such as eCAP or eABR have led to conflicting results. It should be recommended to carry out ICCS assessment prior to evaluate auditory pathway activation. Such an approach is indispensable to better characterize and understand how the spread of CI stimulation may affect the integration of electrical signals provided to auditory neurons.

## 5. Conclusions

At the time of CI fitting, data collected for impedance measurement provide useful information that has been underused so far. The present study showed how voltage matrices can be transformed into a global index of ICCS in the vicinity of the electrode array, upstream of the auditory neurons. Furthermore, ICCS was correlated to speech perception performance in experienced adult CI users.

## Figures and Tables

**Table 1:** Subjects’ characteristics and characteristic values (DU: Duration of CI use, NIE: Number of inactivated electrodes, ESC: Exponential spread coefficient, I40: Intelligibility at 40 dB HL, SRT50: 50% Speech reception threshold—Grey background: no computable SRT50).

Subject	Sex	Aetiology	Implanted Side	DU (Months)	NIE	Reason of Inactivation	ESC	I40 (%)	SRT50 (dB)
S-01	male	Pre lingual, Progressive	Right	24	0		−0.0686	70	33.33
S-02	female	Pre lingual, Progressive, Meningitis	Right	15	0		−0.0677	60	38.33
S-03	female	Post lingual, Progressive, Genetic	Right	27	0		−0.1032	90	26.25
S-04	female	Pre lingual, Congenital, Genetic	Left	17	0		−0.0985	80	24.00
S-05	male	Post lingual, Meniere syndrome	Right	12	0		−0.1030	70	35.00
S-06	male	Pre lingual, Usher	Right	73	0		−0.0804	10	NA
S-07	female	Post lingual, Progressive	Right	25	3	Extra-cochlear	−0.1193	20	44.29
S-08	female	Post lingual, Progressive	Right	20	1	Extra-cochlear	−0.1069	80	27.78
S-09	female	Post lingual, Usher	Right	81	2	Extra-cochlear	−0.0739	70	37.14
S-10	female	Post lingual, Progressive, Genetic	Left	25	4	Non auditory side effect (3 electrodes) or Extra-cochlear	−0.0931	0	58.33
S-11	female	Pre lingual, Progressive	Right	25	0		−0.0746	60	38.33
S-12	female	Post lingual, Ototoxicity	Right	18	0		−0.0650	20	53.33
S-13	female	Post lingual, Ototoxicity	Right	47	0		−0.0618	10	45.00
S-14	male	Post lingual, Progressive	Left	129	2	No auditory percept	−0.0923	50	40.00
S-15	female	Post lingual, Progressive	Right	23	0		−0.0722	30	45.00
S-16	female	Post lingual, Progressive	Right	56	0		−0.0657	40	42.50
S-17	female	Post lingual, Genetic	Left	34	0		−0.0830	70	37.14
S-18	male	Pre lingual, Congenital, Progressive	Right	22	0		−0.0929	40	42.00
S-19	male	Pre lingual, Congenital	Left	23	0		−0.0795	30	46.67
S-20	female	Post lingual, Genetic	Right	12	2	Extra-cochlear	−0.0928	40	42.50
S-21	female	Post lingual	Right	11	0		−0.0933	70	37.14
S-22	female	Post lingual, Progressive	Left	72	1	No auditory percept	−0.0904	40	42.00
S-23	male	Pre lingual	Left	123	1	Non auditory side effect	−0.0629	60	38.33
S-24	female	Post lingual, Otosclerosis	Left	72	0		−0.0874	100	25.00
S-25	male	Post lingual, Ototoxicity	Right	23	0		−0.0942	100	24.00
S-26	male	Post lingual, Meniere syndrome	Right	18	2	No auditory percept	−0.1094	20	47.50
S-27	female	Post lingual, Autoimmune	Right	28	0		−0.0674	80	35.00
S-28	male	Post lingual, Progressive	Right	47	0		−0.0733	50	40.00
S-29	female	Pre lingual, Congenital	Right	11	0		−0.0743	0	NA
S-30	female	Pre lingual, Progressive	Left	23	0		−0.1057	70	37.14
S-31	male	Post lingual, MELAS Syndrome	Right	12	1	Extra-cochlear	−0.0844	70	38.00
S-32	male	Post lingual, Progressive, Genetic	Left	11	0		−0.0349	80	NA
S-33	female	Post lingual, Progressive	Right	25	0		−0.0834	30	43.33
S-34	male	Post lingual, Progressive	Right	11	2	Extra-cochlear	−0.1099	80	34.00
S-35	male	Pre lingual, Congenital, Connexin 26	Right	23			−0.0808	70	36.00
S-36	female	Post lingual, Sudden hearing loss	Left	23	3	Poor sound quality or No auditory percept (2 electrodes)	−0.1287	30	46.67
